# Incorporation of α2-Plasmin Inhibitor into Fibrin Clots and Its Association with the Clinical Outcome of Acute Ischemic Stroke Patients

**DOI:** 10.3390/biom11030347

**Published:** 2021-02-25

**Authors:** Zsuzsa Bagoly, Barbara Baráth, Rita Orbán-Kálmándi, István Szegedi, Réka Bogáti, Ferenc Sarkady, László Csiba, Éva Katona

**Affiliations:** 1 Division of Clinical Laboratory Science, Department of Laboratory Medicine, Faculty of Medicine, University of Debrecen, 4032 Debrecen, Hungary; bagoly@med.unideb.hu (Z.B.); barath.barbi11@gmail.com (B.B.); kalmandi.rita@med.unideb.hu (R.O.-K.); reka.bogati@outlook.com (R.B.); sarkadyf@gmail.com (F.S.); 2 MTA-DE Cerebrovascular and Neurodegenerative Research Group, University of Debrecen, 4032 Debrecen, Hungary; csiba@med.unideb.hu; 3 Kálmán Laki Doctoral School, University of Debrecen, 4032 Debrecen, Hungary; 4 Department of Neurology, Faculty of Medicine, University of Debrecen, 4032 Debrecen, Hungary; szegedii.istvan@gmail.com; 5 Doctoral School of Neuroscience, University of Debrecen, 4032 Debrecen, Hungary

**Keywords:** α2-plasmin inhibitor, acute ischemic stroke, factor XIII, fibrin(ogen), fibrinolysis, thrombolysis

## Abstract

Cross-linking of α2-plasmin inhibitor (α2-PI) to fibrin by activated factor XIII (FXIIIa) is essential for the inhibition of fibrinolysis. Little is known about the factors modifying α2-PI incorporation into the fibrin clot and whether the extent of incorporation has clinical consequences. Herein we calculated the extent of α2-PI incorporation by measuring α2-PI antigen levels from plasma and serum obtained after clotting the plasma by thrombin and Ca^2+^. The modifying effect of FXIII was studied by spiking of FXIII-A-deficient plasma with purified plasma FXIII. Fibrinogen, FXIII, α2-PI incorporation, in vitro clot-lysis, soluble fibroblast activation protein and α2-PI p.Arg6Trp polymorphism were measured from samples of 57 acute ischemic stroke patients obtained before thrombolysis and of 26 healthy controls. Increasing FXIII levels even at levels above the upper limit of normal increased α2-PI incorporation into the fibrin clot. α2-PI incorporation of controls and patients with good outcomes did not differ significantly (49.4 ± 4.6% vs. 47.4 ± 6.7%, *p* = 1.000), however it was significantly lower in patients suffering post-lysis intracranial hemorrhage (37.3 ± 14.0%, *p* = 0.004). In conclusion, increased FXIII levels resulted in elevated incorporation of α2-PI into fibrin clots. In stroke patients undergoing intravenous thrombolysis treatment, α2-PI incorporation shows an association with the outcome of therapy, particularly with thrombolysis-associated intracranial hemorrhage.

## 1. Introduction

Fibrinolysis is a tightly regulated process, accomplished by a wide range of cofactors, receptors and inhibitors [1]. The conversion of plasminogen to plasmin is a central step of fibrinolysis. The most important activator of the generation of plasmin is tissue plasminogen activator (tPA). This protein is used in a recombinant form (rt-PA) as a pharmacological therapy to dissolve blood clots in patients with acute ischemic stroke (AIS), in a process known as thrombolysis [2,3]. Intravenous thrombolysis using rt-PA can only be successful in a short time-window after the onset of stroke symptoms (3–4.5 h). Although this therapy has been proven to be safe and effective in a number of clinical trials and meta-analyses, it is not a remedy for all [4,5]. In the majority of patients (~50-60%), thrombolysis is less effective due to the failure of recanalization, and clinical improvement will not occur. Moreover, in approximately 6–8% of patients, therapy-associated bleeding complications will take place, which potentially leads to worsening of symptoms and might be fatal in approximately 1% of thrombolysed patients [6]. The outcome and safety of thrombolysis is likely to depend on factors influencing or regulating fibrinolysis, but the exact pathomechanism is largely unknown [7,8,9,10].

In order to prevent the newly formed clot from being dissolved prematurely by fibrinolysis, the process of fibrinolysis is regulated on multiple levels [11]. The inhibition of the fibrinolytic system may occur at the level of plasminogen activation, mainly by plasminogen activator inhibitor 1 (PAI-1), or at the level of plasmin, mainly by α2-plasmin inhibitor (α2-PI) [1]. α2-PI is a serine protease inhibitor, and it forms a stable 1:1 complex with plasmin and neutralizes the activity of the enzyme [12,13]. During clot formation, when factor XIII (FXIII) becomes activated, it cross-links α2-PI into the fibrin clot, where it can directly and effectively inhibit plasmin [11,14,15,16]. Little is known about the factors modifying α2-PI incorporation into the fibrin clot and whether the extent of incorporation has clinical consequences. We can surmise that the extent of α2-PI incorporation into the fibrin clot is regulated by a number of factors, such as the heterogeneity of α2-PI and the cross-linking activity of FXIII. In circulation, the full-length α2-PI is proteolytically modified, leading to a variety of circulating α2-PI molecules with modified activities [13]. Approximately 70% of circulating α2-PI is proteolytically cleaved N-terminally [17,18]. The enzyme responsible for the N-terminal cleavage has been purified and identified as antiplasmin cleaving enzyme (APCE, also known as soluble fibroblast activation protein—sFAP) [17]. The N-terminal cleavage has been shown to greatly affect the ability of α2-PI to become cross-linked to the newly formed fibrin clot by activated FXIII (FXIIIa) [14,15,19]. The N-terminal cleavage of α2-PI is affected by a common polymorphism (p.Arg6Trp, rs2070863) [20].

In addition to the N-terminus, α2-PI is post-translationally cleaved at the C-terminus of the molecule. The uncleaved, longer form that can bind plasminogen (PB-α2-PI) comprises about 65% of circulating α2-PI, while the remaining 35% of α2-PI does not bind to plasminogen (NPB-α2-PI) [21]. The enzyme responsible for post-translational cleavage of the C-terminus has not been identified as yet. It has been shown that crosslinking of α2-PI to fibrin primarily involves PB-α2-PI and the removal of the C-terminus regulates α2-PI activity [13]. If we assume that the N-terminal and C-terminal proteolytic modifications of α2-PI occur independently of each other, approximately 70% × 65% = 45% of plasma α2-PI would be expected to be incorporated into the fibrin clot by FXIIIa. Interestingly, scarce literature is available related to this potentially important regulatory step [16,22,23]. Moreover, the extent of α2-PI incorporation into clots has not been investigated in patient cohorts of thromboembolic conditions, in which it may be associated with clinical outcomes. In an early paper, it has been shown in vitro using purified conditions, that cross-linking of α2-PI stops at about 30% incorporation, whereas in the same experiments the cross-linking of fibronectin reached almost 100% [22]. The authors also published that enhancing FXIIIa concentration above 8% does not change maximal incorporation of α2-PI [16]. Unfortunately, FXIII concentration in the latter study was tested in subphysiological levels only, and no follow-up papers were published using higher, physiological FXIII concentrations in the experiments.

Herein we aimed to test the extent of α2-PI incorporation into fibrin clots by developing a new approach, in which total α2-PI antigen levels from the plasma and from the serum of plasma clots are measured and compared. We studied the modifying effect of FXIII levels on the extent of α2-PI incorporation in a wide-range of FXIII concentrations (0–200%). The potential association between thrombolysis outcome and the extent of α2-PI incorporation into fibrin clots obtained from the plasma of acute ischemic stroke patients was also investigated. We report that as opposed to early findings, increasing FXIII levels above 8% results in elevated incorporation of α2-PI into fibrin clots, and the maximal extent of α2-PI incorporation is reached at FXIII levels above 100%. In stroke patients, a lower extent of α2-PI incorporation was found to be associated with more severe stroke, the occurrence of thrombolysis-associated intracranial hemorrhage and worse short-term therapeutic outcomes.

## 2. Materials and Methods

### 2.1. α2-Plasmin Inhibitor Incorporation Assay

The extent of α2-PI incorporation into fibrin clots was studied using an in-house sandwich ELISA assay that measures all forms of α2-PI and is not influenced by the presence of plasmin–antiplasmin complexes (reference range of plasma α2-PI: 48-85 mg/L) [24]. Healthy plasma samples (*n* = 10) were clotted using 2 U/mL thrombin (CoaChrom, Maria Enzersdorf, Austria) and 20 mM CaCl_2_. After incubation at 37 °C for 30 min, serum samples, derived from the extrusion of fluid after plasma clotting, were separated by centrifugation (16,100 g, 5 min). α2-PI antigen levels were measured from the plasma samples before clotting and from the obtained serum samples. The extent of α2-PI incorporation into fibrin clots was calculated using the following formula:

α2-PI incorporation (%) = (plasma α2-PI [mg/L]- serum α2-PI [mg/L])/ plasma α2-PI (mg/L) × 100

The effect of thrombin concentration on the extent of α2-PI incorporation and its time dependence was tested using various amounts of thrombin (0.5, 1, 2, and 5 U/mL) and various clotting times (10, 20, 30, 45, 60, and 180 min). The effect of FXIII levels on α2-PI incorporation into the fibrin clot was investigated using a FXIII-deficient plasma sample with normal α2-PI level. FXIII-deficient plasma aliquots were supplemented with various amounts of purified plasma FXIII (FXIII-A_2_B_2_) (2, 5, 10, 15, 20, 25, 30, 35, and 40 mg/L). Purified FXIII was isolated from pooled plasma of healthy individuals as described previously [25]. After FXIII supplementation, plasma samples were clotted using 2 U/mL thrombin and 20 mM CaCl_2_ for 30 min. Total α2-PI antigen levels in the plasma and serum samples were measured as described above. Clots were washed in compact reaction columns with 20 × 500 μL PBS, pH: 7.2 containing 3 mg/mL iodeacetamide and were dissolved in Laemmli buffer containing 5% mercaptoethanol and 8 M urea at room temperature for 20 h. Dissolved clot samples were analyzed by SDS-PAGE on 7.5% polyacrilamide gels. Proteins were transferred to a PVDF membrane (Bio-Rad, Hercules, CA, USA) and immunostained with polyclonal anti-α2-PI antibody (GA2AP-AP, Affinity Biologicals, ON, Canada) that was labeled with horseradish-peroxidase (HRP) and visualized using enhanced chemiluminescence detection (ECL, Thermo Fisher Scientific, Waltham, MA, USA) on X-ray film (GE Healthcare, Chicago, IL, USA).

### 2.2. Patients and Controls

Fifty-eight acute ischemic stroke [26] patients, all within 4.5 h of their symptom onset before i.v. thrombolysis treatment using rt-PA (Alteplase, Boehringer Ingelheim, Germany) and 26 age-matched healthy controls were recruited in the study. AIS patients were enrolled between September 2016–June 2017 in a single Stroke Center (Department of Neurology, Faculty of Medicine, University of Debrecen, Hungary). All patients received i.v. thrombolysis according to current guidelines, inclusion and exclusion criteria were identical to standard eligibility criteria [27]. Patients who also underwent mechanical thrombectomy were excluded from the study. The presence of AIS was diagnosed based on neurological symptoms and non-contrast CT (NCCT) scan and CT angiography (CTA). Patients were grouped according to their short-term outcome based on the change in their functional neurological status (National Institutes of Health Stroke Scale score, NIHSS) as assessed on admission and at 7 days after thrombolysis. A decrease in the NIHSS score by at least 4 points or to 0 by day 7 was defined as a favourable outcome, while an increase in NIHSS score by at least 4 points was defined as a poor outcome [3,10,28,29]. The presence of hemorrhagic transformation (intracranial bleeding) was defined according to ECASS II criteria based on a control NCCT performed at 24 h post-event [30]. The study was approved by the Ethics Committee of the University of Debrecen, Hungary and the Ethics Board of the Medical Research Council of the Hungarian Ministry of Human Capacities, Hungary. The study protocol conformed to the ethical guidelines of the 1975 Declaration of Helsinki. All patients or their relatives provided written informed consent.

### 2.3. Blood Sampling and Laboratory Measurements

Peripheral blood samples were drawn from AIS patients on admission, before the initiation of rt-PA infusion. Routine laboratory examinations (ions, glucose levels, renal and liver function tests) were performed from serum samples according to standard protocols. High-sensitivity C-reactive protein (hsCRP) level was measured by routine method (Roche Diagnostics, Mannheim, Germany). Citrated blood samples were processed within 1 h of blood drawing and were centrifuged twice at 1220× *g* at room temperature for 15 min. Plasma aliquots were labeled with codes and stored at −70 °C until further measurements. Blood drawing and plasma sample storage of age- and sex-matched healthy controls were performed identically to patient samples. All hemostasis tests were performed by investigators blinded to patient/control identification and clinical data.

Functional fibrinogen levels were measured by the Clauss assay using conventional methods. FXIII-A_2_B_2_ antigen levels were measured by an in-house sandwich ELISA [10,31]. The extent of α2-PI incorporation into the plasma clot was measured in AIS patient and control samples as described above using 2 U/mL thrombin concentration and allowing 30 min for clotting. Plasma soluble fibroblast activation protein (sFAP) antigen levels in AIS patients and healthy controls were measured using human FAP DuoSet^®^ ELISA Development kit (R&D System, Abingdon, UK), following the manufacturer’s protocol and according to earlier reports [32]. The assay was calibrated using recombinant human FAP.

An in vitro clot lysis assay was performed using the platelet-poor plasma of AIS patients and healthy controls. Briefly, a clot induction and lysis mix were prepared, where citrated plasma was mixed with 1000-fold diluted human tissue factor (Innovin, Siemens, Marburg, Germany) and 100 ng/mL rt-PA (Alteplase, Boehringer Ingelheim, Ingelheim, Germany) in HEPES buffer (10mM HEPES, 150 mM NaCl, 0.05% Tween20, pH: 7.4). Clotting and subsequent lysis were induced with automated sample pipetting of HEPES buffer, containing 21 mM CaCl_2_, to each sample well. Optical density was measured at 340 nm, 37 °C every minute for 300 min in a TECAN Infinite m200 microplate reader (TECAN Trading AG, Männedorf, Switzerland). All samples were run in quadruplicate. Curves were analyzed using the Shiny app software tool [33]. The time needed to reach 50% clot lysis (50% clot lysis time; 50%CLT) was defined as the time from the midpoint of the clear-to-maximum-turbid transition, which represents clot formation, to the midpoint of the maximum-turbid-to-clear transition representing the clot degradation.

Genomic DNA was extracted from the buffy coat of blood samples according to standard protocols (QUIamp DNA Blood Mini Kit, Quiagen, Hilden, Germany). The α2-PI Arg6Trp (rs2070863) polymorphism was identified by real-time PCR using fluorescence resonance energy transfer detection and melting curve analysis on a LightCycler^®^ 480 instrument (Roche Diagnostics GmbH, Mannheim, Germany; primers are available from the authors upon request).

### 2.4. Statistical Analysis

Statistical analyses were performed using the Statistical Package for the Social Sciences (SPSS 22, Chicago, IL, USA). The Kolmogorov–Smirnov test was performed to assess normality of data. Between-group differences were analyzed by ANOVA with Bonferroni post hoc test or Kruskal–Wallis test with Dunn’s post hoc analysis depending on the distribution of data. Differences between categorical variables were evaluated by the χ^2^ test. Pearson’s correlation coefficient was calculated to study the strength of correlation between continuous variables. A *p*-value of 0.05 or less was considered to indicate statistical significance.

## 3. Results

### 3.1. The Effect of Thrombin Concentration and Time on the Incorporation of α2-PI into Fibrin Clots

In order to determine the extent of α2-PI incorporation into fibrin clots, we developed a simple method based on the quantification of α2-PI by ELISA in plasma and in the serum after clotting the plasma by thrombin. By subtraction, the extent of α2-PI incorporation in healthy control samples was found to be 44.0 ± 4.6% (*n* = 10).

**Figure 1:**
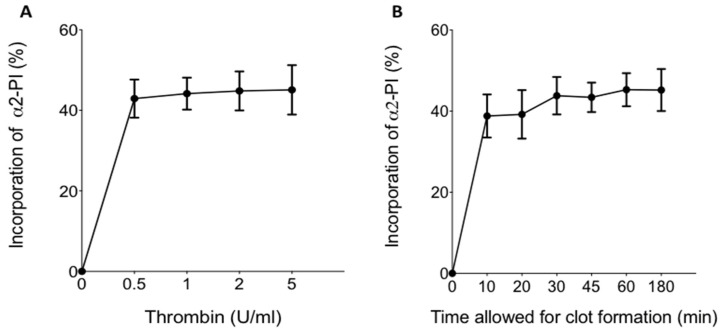
(**A**) Effect of thrombin concentration on the incorporation of α2-plasmin inhibitor (α2-PI) into fibrin clots.; (**B**) time dependence of α2-PI incorporation into fibrin clot. Dots and whiskers indicate mean and standard deviation of the results of 10 parallel experiments.

The effect of thrombin concentration on the extent of α2-PI incorporation and its time dependence was tested using various amounts of thrombin (Figure 1A) and various times allowed for clot formation (Figure 1B). The maximum extent of α2-PI incorporation was found to be approximately 45%. This level of incorporation was reached at relatively low thrombin concentrations (0.5–2 U/mL), and higher thrombin concentrations had no additional effect (Figure 1A). Incorporation of α2-PI into fibrin clots occurred relatively quickly in the presence of 2 U/mL thrombin (Figure 1B). The extent of incorporation was already around 40% after 10 min, and the maximum extent of incorporation was reached after 30 min time allowed for clot formation.

### 3.2. The Effect of FXIII Concentration on the Incorporation of α2-PI into Fibrin Clots

**Figure 2:**
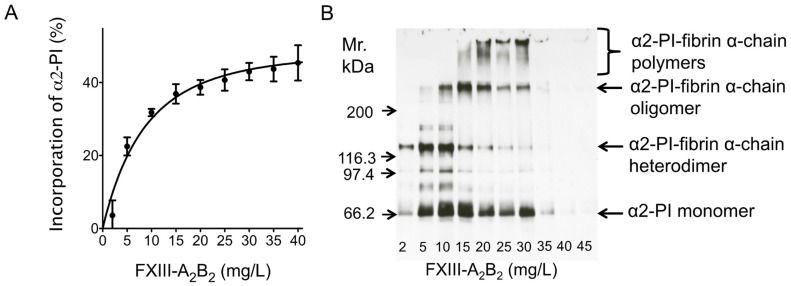
Effect of plasma FXIII level on the amount of α2-PI incorporated into the fibrin clot. A FXIII-deficient plasma sample (α2-PI = 62 mg/L) was supplemented with different amounts of purified FXIII and clotted by thrombin and Ca^2+^. (**A**) Differences of α2-PI antigen values between the substituted plasma and the respective serum were calculated and incorporation was presented as a percentage of the respective plasma value. (**B**) Fibrin clots were washed, dissolved and analyzed by SDS PAGE and Western blot using horseradish-peroxidase (HRP)-labeled polyclonal anti-α2-PI antibody and enhanced chemiluminescence (ECL) detection.

The effect of FXIII levels on the incorporation of α2-PI into fibrin clots was tested using FXIII-deficient plasma supplemented with various amounts of purified FXIII-A_2_B_2_ (Figure 2A,B). As opposed to early reports, where the effect of very low amounts of FXIII was studied on the extent of α2-PI incorporation, here we show that increasing the amounts of FXIII above 8% (mg/L) indeed has additional effect on the extent of α2-PI incorporation. Submaximal extent of incorporation (~40%) was reached in the presence of 21 mg/L (corresponding to 100%) FXIII-A_2_B_2_. Noticeably, increasing FXIII concentrations above this level had a minor additional effect on the extent of α2-PI incorporation into fibrin clots. The effect of FXIII levels on the extent of α2-PI incorporation into fibrin clots was also investigated by SDS PAGE and Western blotting for α2-PI, after washing and dissolving the fibrin clots (Figure 2B). Using this approach, it became evident that by increasing the concentration of FXIII in the plasma samples, the amount of cross-linked α2-PI-fibrin α-chain polymers increase within the fibrin clots. This was detectable at concentrations of up to 30 mg/L FXIII. On the other hand, increasing FXIII concentration above this level resulted in highly crosslinked fibrin clots that could not be dissolved using standard Laemmli buffer and therefore could not be investigated using this approach.

### 3.3. The Extent of α2-PI Incorporation into Plasma Clots in Acute Ischemic Stroke Patients and Its Relation to Thrombolysis Outcome

In order to investigate whether the extent of α2-PI incorporation into plasma clots is associated with thrombolysis outcome in acute stroke patients, the levels of fibrinogen, FXIII-A_2_B_2_ and the extent of and α2-PI incorporation were measured from the plasma samples of 57 acute ischemic stroke patients, all within 4.5 h of their symptom onset before intravenous thrombolysis treatment and 26 age-matched healthy controls (Table 1). Patients were grouped according to their short-term outcome. Patients experiencing therapy-associated intracranial hemorrhage were significantly older and had significantly more severe stroke based on admission NIHSS as compared to those with good outcome. FXIII levels were significantly lower in all patient groups as compared to healthy controls. Fibrinogen levels were significantly increased in patients with therapy failure (no change/poor outcome) as compared to healthy controls. It must be noted that admission CRP levels were significantly increased in patients with no change/poor outcomes and in patients with therapy-associated intracranial hemorrhage as compared to healthy controls. In patients with post-lysis intracranial hemorrhage, plasma α2-PI levels were significantly lower as compared to healthy controls.

**Figure 3:**
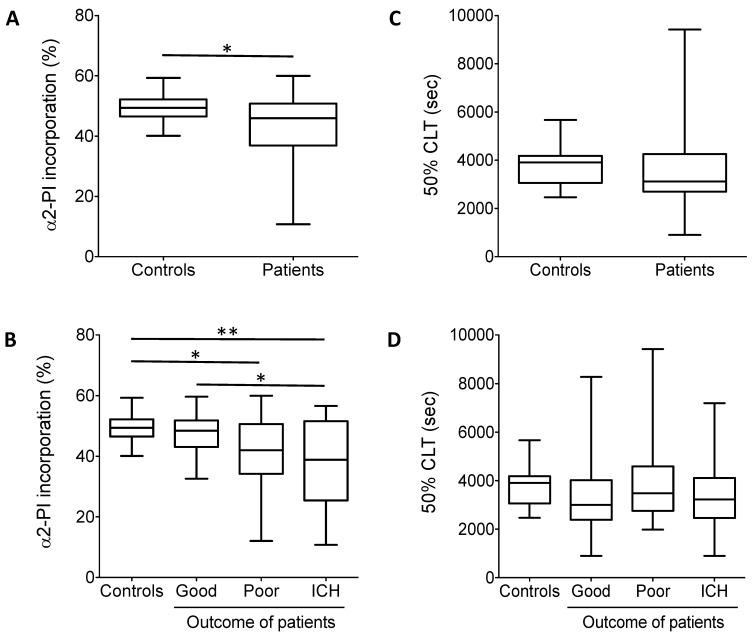
(**A**) α2-PI incorporation into the fibrin clot. Plasma samples were clotted by thrombin and Ca^2+^. Differences between plasma and serum α2-PI antigen values were calculated and incorporation was presented as percentage of the respective plasma values in controls and in the total cohort of patients. (**B**) α2-PI incorporation into the fibrin clot in controls and in patients according to different outcomes. (**C**) In vitro clot lysis experiments. The 50% CLT parameter is shown in controls and in the total cohort of patients. The 50% clot lysis times (CLTs) did not show significant differences between controls and patients. (**D**) In vitro clot lysis experiments in controls and in patients according to different outcomes. The 50% CLT did not show significant differences among patient groups. Box and whisker plots indicate median, interquartile range, and total range *, *p* < 0.05; **, *p* < 0.01.

The extent of α2-PI incorporation into fibrin clots was significantly lower in the total cohort of patients as compared to healthy controls (Figure 3A). When patients were grouped according to thrombolysis outcomes, the extent of α2-PI incorporation was found to be significantly lower in patients with no change/poor outcomes and patients with post-lysis intracranial hemorrhage (41.5 ± 11.8% and 37.3 ± 14.0%, respectively) as compared to healthy controls (49.4 ± 4.6%) (Figure 3B). The extent of α2-PI incorporation in patients with good outcome (47.4 ± 6.7%) did not differ significantly from that observed in controls. On the other hand, the extent of α2-PI incorporation was significantly lower in those who suffered post-lysis intracerebral hemorrhage as compared to those with good outcomes (Figure 3B).

**Figure 4:**
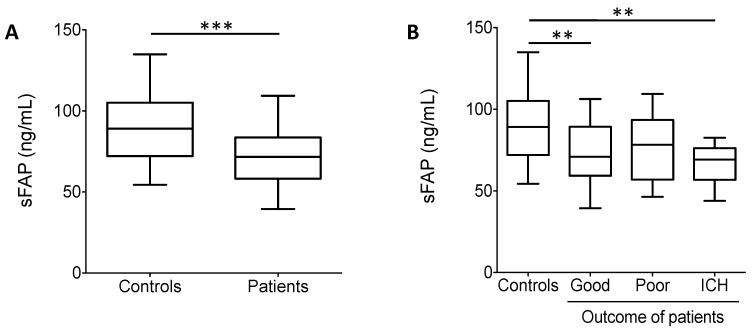
(**A**) Soluble fibroblast activation protein (sFAP) levels in controls and in the total cohort of patients. sFAP levels were significantly lower in patients as compared to healthy controls. (**B**) sFAP levels in controls and in patients according to different outcomes. Box and whisker plots indicate median, interquartile range, and total range. **, *p* < 0.01, ***, *p* < 0.001.

In vitro clot lysis (Figure 3C,D) and additional parameters influencing α2-PI heterogeneity (sFAP and α2-PI p.Arg6Trp polymorphism) were also determined from the plasma samples of patients and controls (Figure 4A,B and Table 1). Median time to reach 50% clot lysis did not differ significantly between controls and patients (Figure 3C), moreover, 50%CLT did not show significant differences between controls and patients with different outcomes. On the other hand, sFAP levels were significantly lower in patients as compared to healthy controls (Figure 4A). In particular, the level of sFAP was significantly lower in those who suffered post-lysis intracerebral bleeding and in those with good outcome as compared to healthy controls (Figure 4B). α2-PI p.Arg6Trp polymorphism had no influence on outcomes (Table 1) and its allele frequency was essentially the same in controls and patients. α2-PI p.Arg6Trp polymorphism had no influence on the extent of α2-PI incorporation to fibrin clots in either groups (data not shown).

When looking at the correlations between various baseline parameters and the extent of α2-PI incorporation into fibrin clot, in controls, fibrinogen levels showed a significant positive correlation with the extent of α2-PI incorporation (Table 2). In patients, a significant positive correlation was found between plasma α2-PI levels, FXIII levels and the extent of α2-PI incorporation into fibrin clots. Moreover, a highly significant negative association was found between NIHSS on admission in patients and the extent of α2-PI incorporation. This suggests that in case of more severe strokes, less α2-PI is incorporated into fibrin clots in vitro. As the extent of α2-PI incorporation has its limit (45–50%), the most likely reason for this association is that the fraction of α2-PI that could be incorporated into fibrin clots is less available in the plasma samples of patients with more severe strokes due to considerable in vivo consumption. The negative association between NIHSS and the extent of α2-PI incorporation was particularly strong in the subgroup of patients with post-lysis intracerebral hemorrhage (r = −0.627, *p* = 0.039)

## 4. Discussion

It has been shown by elegant studies that the stability of the newly formed thrombi against premature fibrinolysis predominantly depends on the cross-linking of α2-PI into the fibrin clot [34,35]. Despite the great importance of this regulatory step and its potential influence on a wide range of thrombotic clinical events, surprisingly little is known on the extent of α2-PI incorporation into the fibrin clot under various circumstances. Herein we developed a new approach to test the extent of α2-PI incorporation into fibrin clots by measuring total α2-PI antigen levels from the plasma and the serum of plasma clots obtained after clotting. Using this method, the extent of α2-PI incorporation in healthy control samples was found to be 44.0 ± 4.6%, consistent with the calculated estimated amount of potentially cross-linked α2-PI, based on the ratio of isoforms that are efficiently cross-linked by FXIIIa. In earlier studies, different approaches were used to detect α2-PI in clots. Using SDS-PAGE and Western blotting of fibrin clots made from purified proteins to follow α2-PI-fibrin α-chain crosslinking, approximately 30% of α2-PI was reported to be incorporated into fibrin [17,22]. A limitation of such studies is that purified α2-PI used in the experiments was comprised of the plasminogen-binding form only. Moreover, Western blotting is not an optimal approach to quantify the amount of highly crosslinked α2-PI-fibrin α-chain polymers. In a more recent study, a similar approach to our method was used. The quantification of clot-incorporated α2-PI was performed by ELISA in plasma and in the serum after thrombus formation, however, in that case thrombus formation was followed in a Chandler loop. By subtraction, the extent of incorporation into thrombi was found to be between 30–50% [35]. Our method is less laborious as compared to thrombus formation in a Chandler loop, which allowed us to perform the investigations in a cohort of AIS patients and healthy controls.

The extent of incorporation is not only the result of FXIIIa-mediated cross-linking of α2-PI to fibrin, but to a somewhat lesser extent, the result of a non-covalent binding of α2-PI to fibrin [13,36]. The latter interaction has been implicated to potentially contribute to the proper orientation of α2-PI and thus facilitate the cross-linking process [36]. Here we provide evidence that as opposed to early findings [16], enhancing FXIII concentration above 8% results in elevated incorporation of α2-PI into fibrin clots, and the maximal extent of α2-PI incorporation is reached at FXIII levels above 100%. By studying the modifying effect of FXIII levels on the extent of α2-PI incorporation in a wide-range of FXIII concentrations (0–200%), we were able to demonstrate that increasing FXIII levels up to supraphysiological levels result in gradual, extensive cross-linking of α2-PI to the fibrin clot. This might represent a missing biochemical link related to clinical observations on the role of FXIII in acute thrombotic events. It has been published that elevated FXIII levels increase the risk of myocardial infarction in young adults and particularly in women [37,38,39]. Reduced fibrinolytic capacity has been defined as a risk factor for myocardial infarction and stroke in young patients, but the exact mechanisms have not been identified as yet [40,41]. It has been presumed that elevated FXIII, by extensively cross-linking α2-PI to the fibrin clot and effectively inhibiting fibrinolysis, could play a role in sustaining the occluding thrombus in circumstances when atherosclerosis is not as prominent [38].

During thrombolytic treatment, regulatory steps inhibiting fibrinolysis must be overcome to achieve effective break-down of thrombi. It has been shown in animal models that the resistance of thrombi to t-PA-induced thrombolysis is greatly influenced by the amount of active α2-PI contained in the newly formed thrombi [42,43,44,45]. Inhibition of clot-bound α2-PI enhanced thrombolysis significantly in a rabbit jugular vein thrombosis model [42], and treating mice with an α2-PI-inactivating antibody resulted in quick thrombus dissolution [43]. Effects of elevated α2-PI levels and its inhibition have been extensively studied in mice models of stroke thrombolysis and based on the results, proposals to enhance thrombolysis in acute stroke patients by inhibiting clot-bound α2-PI have been suggested [42,44,45]. Based on data derived from animal models, the potential association between thrombolysis outcome and the extent of α2-PI incorporation into clots obtained from the plasma of acute ischemic stroke patients is intriguing. Despite our knowledge derived from experimental stroke models, surprisingly, the extent of α2-PI incorporation into clots has not been yet investigated in acute stroke patient cohorts undergoing thrombolysis, where it may be associated with clinical outcomes. Here we show that in acute stroke patients within 4.5 h of their symptom onset, lower extent of in vitro α2-PI incorporation was found in those with more severe stroke. Our results demonstrated a highly significant negative association between NIHSS on admission and the extent of α2-PI incorporation. These results might be explained by a potential, considerable consumption of the fraction of α2-PI that can be incorporated into the clot. As the extent of α2-PI incorporation has its limit (45–50%), we can surmise that the fraction of α2-PI that could be incorporated into fibrin clots is less available in the plasma samples of patients with more severe strokes due to significant in vivo consumption. It has been known for a long time that more severe strokes are associated with less favorable thrombolysis outcomes and lysis resistance, together with a higher chance of bleeding complications, but the exact reason for these associations has not been clarified [46]. Here we show that the extent of in vitro α2-PI incorporation into plasma clots was significantly lower in patients who suffered post-lysis intracranial hemorrhage as compared to those with favorable outcome. Similarly to what has been known from the literature [47,48], patients with therapy-associated intracranial hemorrhage presented with a significantly more severe stroke on admission (higher NIHSS) in our cohort. More severe strokes might be associated with larger thrombus burden, higher extent of α2-PI incorporation and thus lower susceptibility to thrombolysis together with an increased risk of bleeding. In these cases, a lower extent of α2-PI incorporation in vitro could implicate that in vivo the balance of fibrinolysis might be shifted towards bleeding at the surrounding sites of the thrombus. Due to the lower availability of clot-bound α2-PI, protection against t-PA-induced hemostatic challenge might be diminished in these cases. Post-lysis intracerebral hemorrhage is a feared side effect of rt-PA induced thrombolysis, and despite its adverse effects on clinical outcomes, little is known about the exact pathomechanism as yet [8,46,49]. Experimental results derived from patients’ samples on admission showing association with future intracerebral bleeding events are potentially helpful to understand the background of bleeding events and to implement strategies for the prevention of such events.

In this study, parameters influencing α2-PI heterogeneity (sFAP and α2-PI p.Arg6Trp polymorphism) were tested but we could not show an association between these factors and clinical outcomes. Similarly to other studies in patients suffering from arterial thrombosis (coronary heart disease, ischemic stroke or peripheral arterial disease) [13,32,50], sFAP levels were significantly lower in this cohort of acute stroke patients as compared to controls. Here we show that the α2-PI p.Arg6Trp polymorphism had no influence on thrombolysis outcomes and its allele frequency was essentially the same in controls and patients in this cohort. Only few studies have investigated the α2-PI p.Arg6Trp polymorphism in relation to the risk of thrombotic events [13]. These studies were inconclusive or could not show an effect of the α2-PI p.Arg6Trp polymorphism on the risk of arterial thrombotic events, while outcomes have not been investigated. In the future, well-designed prospective studies are warranted to investigate the role of such parameters influencing α2-PI heterogeneity in relation to the risk of arterial thrombotic events and outcomes.

## 5. Conclusions

Increased FXIII levels result in elevated incorporation of α2-PI into fibrin clots. In acute stroke patients undergoing intravenous thrombolysis treatment, the extent of α2-PI incorporation shows an association with the outcome of therapy, particularly with the occurrence of thrombolysis-associated intracranial hemorrhage.

## Figures and Tables

**Table 1:** Characteristics of the study population.

	Control (*n* = 26)	Good Outcome (*n* = 25)	No Change/Poor Outcome (*n* = 20)	Therapy Associated Intracranial Hemorrhage (*n* = 12)
Age (years)	66.4 ± 5.6	61.4 ± 13.6	67.5 ± 12.6	72.2 ± 10.0 ^§§^
Gender (F/M)	12/14	12/13	8/12	7/5
Hypertension (no/yes)	13/13	5/16	2/15 *	4/6
Smoking (no/yes)	21/5	16/5	12/5	7/3
BMI	27.1 ± 4.2	28.4 ± 4.8	27.6 ± 4.1	26.4 ± 4.9
NIHSS on admission	-	4.0 (3.0–7.0)	9 (5–11.5)	10.0 (5.0–16.0) ^§^
NIHSS at 7 days after thombolysis	-	1.0 (0.0–2.0)	11.0 (6.5–16.0) ^§§§^	10 (6.5–11.0) ^§§§^
CRP (mg/L)	1.7 (0.8–3.4)	2.9 (1.4–5.1)	4.9 (2.8–7.1) **	5.3 (2.5–11.9) **
Fibrinogen (g/L)	3.6 ± 0.5	3.9 ± 1.3	4.3 ± 1.5 *	4.2 ± 2.0
FXIII-A_2_B_2_ antigen (mg/L)	28.2 ± 4.3	23.4 ± 5.5 *	22.9 ± 7.6*	21.3 ± 6.7 **
Plasma α2-PI (mg/L)	66.9 ± 8.7	64.9 ± 10.8	56.8 ± 12.8	52.8 ± 19.7 *^§^
Serum α2-PI (mg/L)	33.9 ± 4.2	34.1 ± 6.1	32.2 ± 5.9	32.2 ± 13.3
α2-PI p.Arg6Trp (Wild type/Carrier)	15/5	17/8	13/7	6/6

Values of continuous variables are presented as mean ± SD and (median (IQR) where appropriate. Abbreviations: F, female; M, male; BMI, body mass index; NIHSS, The National Institutes of Health Stroke Scale; CRP, C-reactive protein; α2-PI, α2-plasmin inhibitor; *, *p* < 0.05 Patient group vs. Control; **, *p* < 0.01 Patient group vs. Control; ^§^, *p* < 0.05 Patient group vs. Good outcome; ^§§,^ *p* < 0.01 Patient group vs. Good outcome; ^§§§,^ *p* < 0.01 Patient group vs. Good outcome.

**Table 2:** Correlation of different parameters with the extent of α2-PI incorporation into fibrin clots.

	Control (*n* = 26)	Patients (*n* = 57)	Total (*n* = 83)
Age	−0.051 (0.803)	−0.224 (0.093)	−0.200 (0.070)
Fibrinogen	0.453 (0.039)	0.224 (0.098)	0.171 (0.137)
FXIII-A_2_B_2_	0.101 (0.622)	0.303 (0.022)	0.357 (0.001)
Plasma α2-PI	0.284 (0.160)	0.544 (<0.001)	0.540 (<0.001)
NIHSS on admission	-	−0.449 (0.001)	

Values represent Pearson’s correlation coefficient and statistical significance, r (*p*).

## Data Availability

The data presented in this study are available on request from the corresponding author.
